# How age and sex affect the erythrocyte sedimentation rate and C-reactive protein in early rheumatoid arthritis

**DOI:** 10.1186/1471-2474-15-368

**Published:** 2014-11-06

**Authors:** Liseth Siemons, Peter M ten Klooster, Harald E Vonkeman, Piet LCM van Riel, Cees AW Glas, Mart AFJ van de Laar

**Affiliations:** Arthritis Center Twente, Department of Psychology, Health & Technology, University of Twente, Enschede, The Netherlands; Arthritis Center Twente, Department of Rheumatology, Medisch Spectrum Twente, Enschede, The Netherlands; Scientific Institute for Quality of Healthcare, Radboud University, Nijmegen, The Netherlands; Department of Research Methodology, Measurement and Data Analysis, University of Twente, Enschede, The Netherlands; Department of Psychology, Health & Technology, Faculty of Behavioural Sciences, University of Twente, PO Box 217, 7500 AE Enschede, The Netherlands

**Keywords:** Erythrocyte sedimentation rate (ESR), C-reactive protein (CRP), Age, sex, body mass index (BMI), Early rheumatoid arthritis (RA)

## Abstract

**Background:**

The erythrocyte sedimentation rate (ESR) and C-reactive protein (CRP) are two commonly used measures of inflammation in rheumatoid arthritis (RA). As current RA treatment guidelines strongly emphasize early and aggressive treatment aiming at fast remission, optimal measurement of inflammation becomes increasingly important. Dependencies with age, sex, and body mass index have been shown for both inflammatory markers, yet it remains unclear which inflammatory marker is affected least by these effects in patients with early RA.

**Methods:**

Baseline data from 589 patients from the DREAM registry were used for analyses. Associations between the inflammatory markers and age, sex, and BMI were evaluated first using univariate linear regression analyses. Next, it was tested whether these associations were independent of a patient’s current disease activity as well as of each other using multiple linear regression analyses with backward elimination. The strengths of the associations were compared using standardized beta (β) coefficients. The multivariate analyses were repeated after 1 year.

**Results:**

At baseline, both the ESR and CRP were univariately associated with age, sex, and BMI, although the association with BMI disappeared in multivariate analyses. ESR and CRP levels significantly increased with age (β-ESR = 0.017, *p* < 0.001 and β-CRP = 0.009, *p* = 0.006), independent of the number of tender and swollen joints, general health, and sex. For each decade of aging, ESR and CRP levels became 1.19 and 1.09 times higher, respectively. Furthermore, women demonstrated average ESR levels that were 1.22 times higher than that of men (β = 0.198, *p* = 0.007), whereas men had 1.20 times higher CRP levels (β = -0.182, *p* = 0.048). Effects were strongest on the ESR. BMI became significantly associated with both inflammatory markers after 1 year, showing higher levels with increasing weight. Age continued to be significantly associated, whereas sex remained only associated with the ESR level.

**Conclusions:**

Age and sex are independently associated with the levels of both acute phase reactants in early RA, emphasizing the need to take these external factors into account when interpreting disease activity measures. BMI appears to become more relevant at later stages of the disease.

## Background

Acute phase reactants are commonly used as a measure of inflammation in rheumatoid arthritis (RA) and are part of the provisional definition of RA remission as defined by the ACR/EULAR [1], the ACR preliminary core set of RA disease activity measures [2], and the 28-joint Disease Activity Score (DAS28) [3]. Traditionally, the erythrocyte sedimentation rate (ESR) has been the most widely used marker of inflammation in RA. The ESR is an indirect measure of inflammation, which reflects the level of acute-phase plasma proteins in the blood (e.g. fibrinogen) because these cause the red blood cells to settle more rapidly [4]. However, a number of limitations of this inflammatory marker have become apparent over the years. Although the test is relatively easy and inexpensive to perform, ESR levels respond slowly to inflammatory stimuli and, thus, to changes in disease activity. Given the current emphasis on strict and aggressive treatment strategies [5, 6] that suppress RA disease activity as early, fast and complete as possible [7, 8], this slow response of the ESR levels is often considered a limitation. Also, because the ESR is a non-specific acute phase reactant of systemic inflammation, elevated levels are not necessarily (solely) due to the inflammation of the rheumatic disease. It has been shown that ESR levels can be greatly influenced by, for instance, infections, malignancies, abnormally shaped or sized red blood cells or serum protein concentrations [9]. They also tend to be higher in females than in males [10–15] and appear to increase with age [10–17] and with body mass index (BMI) [4, 13, 18]. Thus, even though the ESR is still widely used in clinical research and practice because of its familiarity, its simplicity, and the attention it received over the years [9], these limitations may complicate the use of the ESR in assessing RA disease activity.

In an attempt to overcome some of these limitations, the C-reactive protein (CRP) has been suggested as an alternative inflammatory marker of disease activity in RA [19]. The CRP is a protein that is produced in the liver as a reaction to certain biologic ligands that appear when inflammation develops [4]. Many studies tend to favor CRP over ESR in assessing RA inflammation [20], as it is believed to give a better reflection of current disease activity than ESR because of its more rapid response to increases or decreases in inflammatory stimuli [4, 9]. Another commonly supposed advantage of CRP is its lower susceptibility to external confounding factors like age and sex, compared to the ESR [4]. However, an extensive number of studies have suggested that CRP may exhibit similar dependencies on age [16, 21–23], sex [22–24], and BMI [4, 18, 21–23, 25–27].

As current RA treatment guidelines strongly emphasize early and aggressive treatment aiming at fast remission [5, 6], optimal measurement of inflammation in this patient group is becoming increasingly important. To date, however, it remains unclear which inflammatory marker is affected most strongly by the external effects of age, sex, and BMI in patients with early RA and with this study we aim to provide more insight into these associations.

## Methods

### Patients

Data were used from the remission induction cohort of the Dutch Rheumatoid Arthritis Monitoring (DREAM) registry [28]. This observational multicenter cohort included patients that were newly and clinically diagnosed with RA by a certified rheumatologist, who experienced symptoms for less than a year and who were not in remission at the time of inclusion. Patients followed a treat-to-target treatment protocol that aimed at a fast remission (DAS28 < 2.6). This protocol has been described elsewhere [28, 29]. Only patients who had a valid baseline measure of both the ESR and the CRP were included for analyses, which resulted in the exclusion of 37 patients. Independent t‒tests, Kruskal Wallis tests, and Chi‒Square tests showed that the excluded patients did not differ from the other patients in any of the collected baseline measures (described in the next paragraph). All patients were 18 years or older and had not used DMARDs or prednisolone before. Patient recruitment took place from 2006 to 2012 and data collection of included patients is still ongoing. Informed consent was obtained from each patient. No ethical approval was required, as evaluated by the ethics committees of the participating hospitals and in accordance with Dutch law, since all data collected are part of daily clinical practice.

### Measures

Baseline data were collected on several demographic, clinical, and patient-reported measures. The patient’s age, sex, anti-CCP positivity, and rheumatoid factor were registered, BMI was calculated as the ratio of weight in kilograms and the square of the height in meters, physical functioning was assessed with the Health Assessment Questionnaire (HAQ, score range 0-3, where higher values reflect worse outcomes) [30], and physical and mental health status with the Short Form Health Survey with 36 items (SF-36, score range 0-100, where lower values reflect worse outcomes) [31]. At each visit, disease activity was assessed by a trained rheumatologist or nurse practitioner following a standardized procedure using either the DAS28-ESR or the DAS28-CRP, respectively [3, 19]. The DAS28 is an index measure including a 28-joint count of both tenderness and swelling (TJC28 and SJC28, score range 0-28), a patient reported visual analog scale of general health (GH, score range 0-100), and an inflammatory marker, which can be either the ESR or the CRP.

### Statistical analysis

BMI was divided into 3 categories (i.e. normal weight (<25 kg/m^2^), overweight (25-30 kg/m^2^), and obese (>30 kg/m^2^)) and the values of the ESR and CRP were natural-log-transformed to normalize their distributions. The associations between the inflammatory markers and age, sex, and BMI were evaluated using univariate linear regression analyses first (using *p* < 0.10 for significance). Next, it was tested whether these associations were independent of a patient’s current disease activity (as measured with the other core components of the DAS28) as well as of each other by performing multiple linear regression analyses with backward elimination. Possible interaction terms of the remaining variables were tested for significance. Standardized beta coefficients were used to compare the strength of the relationships in the final models. To evaluate whether the associations changed over time, the multivariate analyses were repeated on the 1-year’s data (i.e. data collected after 1 year of treatment). All analyses were performed using SPSS, version 21.0. Statistical significance in the multivariate analyses was defined as *p* < 0.05.

## Results

### Patients

A total sample of 589 early RA patients was included for analyses. At inclusion, patients were not in remission as characterized by a DAS28 score >2.6 (i.e. the cut-off point for not being in remission), several tender and swollen joints, and a diminished feeling of general and physical health (Table 1). Patients were on average 57 years old, the majority being female, and most had some degree of overweight (60.7% had a BMI ≥25 kg/m^2^).

**Table 1:** **Characteristics of the study population at inclusion**

Characteristic	Mean (SD) or Median (IQR)*
Sex (female)	362/589 (61.5%)
Age (years)	57.34 (14.20)
Symptom duration (weeks)	14.00 (8.00-26.00)
Body mass index (kg/m^2^)	26.59 (4.44)
Normal (N = 198)	22.64 (1.89)
Overweight (N = 209)	27.09 (1.35)
Obese (N = 97)	33.54 (3.23)
ESR (mm/hour)	21.00 (11.00-36.00)
CRP (mg/l)	7.00 (5.00-19.50)
28-Tender joint count	3.00 (0.00-7.00)
28-Swollen joint count	5.00 (2.00-9.00)
General health	44.55 (26.39)
DAS28-ESR	4.23 (1.51)
DAS28-CRP	4.00 (1.35)
SF36 – physical health	37.41 (9.32)
SF36 – mental health	48.13 (11.59)
HAQ	0.99 (0.72)
Anti-CCP positive	139/228 (61.0%)
Rheumatoid factor positive	285/520 (54.8%)

*The values for sex, RF and anti-CCP positivity are the number of patients/number of patients assessed. DAS-28 = disease activity score for 28 joints, ESR = erythrocyte sedimentation rate, CRP = C-Reactive Protein, SF36 = Short Form Health Survey with 36 items, HAQ = Health Assessment Questionnaire.

### Univariate analyses

Without controlling for possible confounding factors, both ESR and CRP levels increased with age and tended to increase with BMI (Table 2). Furthermore, women tended to have higher ESR levels than men, whereas men tended to have higher CRP levels.

**Table 2:** **Univariate associations of the inflammatory markers (ESR and CRP) with age, sex, and BMI at baseline**

Univariate association*	Unstandardized coefficient		Standardized coefficient	***p***-value
	Beta (95%CI)	Standard error	Beta (95%CI)	
**ESR**				
Age	0.019 (0.014 - 0.024)	0.003	0.296 (0.219 – 0.373)	<0.001
Female sex	0.136 (-0.018 - 0.290)	0.078	0.072 (-0.009 – 0.152)	0.083
Overweight	0.048 (-0.126 - 0.222)	0.088	0.026 (-0.067 – 0.118)	0.587
Obese	0.190 (-0.027 - 0.407)	0.110	0.081 (-0.011 – 0.173)	0.086
**CRP**				
Age	0.013 (0.007-0.020)	0.003	0.164 (0.084 – 0.244)	<0.001
Female sex	-0.183 (-0.372 – 0.006)	0.096	-0.078 (-0.159 – 0.002)	0.057
Overweight	0.248 (0.035 – 0.462)	0.109	0.108 (0.015 – 0.200)	0.023
Obese	0.250 (-0.018 – 0.517)	0.136	0.086 (-0.006 – 0.179)	0.067

*Analyses were performed on the naturally log-transformed ESR and CRP values to normalize their distributions.

ESR = erythrocyte sedimentation rate, CRP = C-Reactive Protein, CI = confidence interval. The males and normal weight patients were used as reference categories.

### Multivariate analyses

#### At baseline

After adjusting for the other core components of disease activity that are included in the DAS28 (i.e. the TJC28, SJC28, and GH), sex and age were still independently associated with both inflammatory markers, while BMI was no longer related to a patient’s ESR or CRP level (Table 3). Although the effects of age were slightly attenuated, the effects of sex were not and became even stronger in the ESR model.

**Table 3:** **Multivariate associations of the inflammatory markers (ESR and CRP) with age and sex at baseline (N = 589)**

Variables*	Unstandardized coefficient		Standardized coefficient	***p***-value
	Beta (95%CI)	Standard error	Beta (95%CI)	
**ESR model**				
TJC28	-0.012 (-0.028 – 0.003)	0.008	-0.075 (-0.168 – 0.018)	0.115
SJC28	0.044 (0.028 – 0.059)	0.008	0.256 (0.166 – 0.345)	<0.001
General health	0.006 (0.003 – 0.009)	0.001	0.170 (0.089 – 0.250)	<0.001
Female sex	0.198 (0.053 – 0.342)	0.073	0.104 (0.028 – 0.179)	0.007
Age	0.017 (0.012 – 0.022)	0.003	0.262 (0.186 – 0.337)	<0.001
**CRP model**				
TJC28	-0.001(-0.020 – 0.018)	0.010	-0.006 (-0.100 – 0.089)	0.908
SJC28	0.058 (0.039 – 0.077)	0.010	0.279 (0.188 – 0.370)	<0.001
General health	0.008 (0.004 – 0.011)	0.002	0.180 (0.098 – 0.262)	<0.001
Female sex	-0.182 (-0.362 – -0.001)	0.092	-0.078 (-0.155 – -0.001)	0.048
Age	0.009 (0.002 – 0.015)	0.003	0.108 (0.031 – 0.185)	0.006

*ESR = erythrocyte sedimentation rate, CRP = C-Reactive Protein, TJC28 = tender joint count in 28-joints, SJC28 = swollen joint count in 28-joints. CI = confidence interval. R^2^ ESR-model: 18.7%, R^2^ CRP model: 16.2%.

Thus, obese patients did not show significantly higher levels of ESR or CRP (mean ± SD ESR: 29.34 ± 21.87, CRP: 17.30 ± 22.06) than overweight (ESR: 26.28 ± 18.76, CRP: 18.14 ± 19.84) or normal weight patients (ESR: 26.70 ± 22.25, CRP: 16.97 ± 24.21). On the other hand, ESR and CRP levels did significantly increase with age (β-ESR = 0.017, *p* < 0.001 and β-CRP = 0.009, *p* = 0.006), independent of the number of tender and swollen joints, the patient-reported degree of general health, and the patient’s sex. The unstandardized betas (0.017 vs. 0.009) represent the change of the natural-log-transformed ESR and CRP levels, respectively, for each year of aging. Transformed back to their normal values, this means that for each decade of aging the ESR and CRP levels become 1.19 and 1.09 times higher, respectively. Furthermore, women demonstrated average ESR levels that were 1.22 times higher than those of the men (β = 0.198, *p* = 0.007), whereas men had 1.20 times higher CRP levels (β = -0.182, *p* = 0.048). The effects of age and sex were strongest in the ESR model, indicating that the ESR is more sensitive to the effects of these external factors. Although there were no significant interactions between age and sex (interaction ESR model: p = 0.222 and interaction CRP model: p = 0.665), differences in ESR and CRP between male and female patients appeared to gradually decrease with age (Table 4).

**Table 4:** **ESR and CRP levels in men and women per age group**

Men (N = 227)		Women (N = 362)	
Variable*	Median (IQR)	Variable*	Median (IQR)
**ESR**		**ESR**	
Age group		Age group	
<55 years (n = 70)	11.00 (4.75-29.00)	<55 years (n = 161)	15.00 (8.00-29.00)
55 - 65 years (n = 80)	17.50 (10.00-33.00)	55 - 65 years (n = 95)	25.00 (12.00-37.00)
>65 years (n = 77)	31.00 (17.50-41.50)	>65 years (n = 106)	27.00 (17.75-42.00)
**CRP**		**CRP**	
Age group		Age group	
<55 years (n = 70)	6.50 (5.00-17.50)	<55 years (n = 161)	5.00 (5.00-13.00)
55 - 65 years (n = 80)	8.50 (5.00-24.00)	55 - 65 years (n = 95)	5.00 (5.00-18.00)
>65 years (n = 77)	11.00 (5.00-26.50)	>65 years (n = 106)	10.00 (5.00-24.25)

*ESR = erythrocyte sedimentation rate, CRP = C-Reactive Protein.

#### After 1 year

Interestingly, BMI did become significantly associated with both the ESR and the CRP after 1 year (Table 5). Heavier patients show higher ESR and CRP levels. Age continued to be significantly associated, although this relationship was no longer linear but increased exponentially. Sex, on the other hand, was still significantly associated with ESR levels (showing higher levels in females), but was no longer significantly associated with CRP (*p* = 0.855).

**Table 5:** **Multivariate associations of the inflammatory markers (ESR and CRP) with age, sex, and BMI after 1 year (N = 365)**

Variables*	Unstandardized coefficient		Standardized coefficient	***p***-value
	Beta (95% CI)	Standard error	Beta (95% CI)	
**ESR model**				
TJC28	-0.024 (-0.056 – 0.008)	0.016	-0.088 (-0.206 – 0.031)	0.147
SJC28	0.014 (-0.030 – 0.058)	0.022	0.035 (-0.074 – 0.144)	0.527
General health	0.004 (0.000 – 0.008)	0.002	0.103 (-0.012 – 0.219)	0.080
Female sex	0.370 (0.187 – 0.552)	0.093	0.213 (0.108 – 0.317)	<0.001
Age: 55-65 years	0.151 (-0.055 – 0.356)	0.105	0.084 (-0.031 – 0.198)	0.151
Age: >65 years	0.570 (0.363 – 0.777)	0.105	0.320 (0.203 – 0.436)	<0.001
Overweight	-0.349 (-0.581 – -0.117)	0.118	-0.207 (-0.345 – -0.069)	0.003
Normal weight	-0.283 (-0.520 – -0.045)	0.121	-0.164 (-0.302 – -0.026)	0.020
**CRP model** ^**#**^				
TJC28	-0.014 (-0.047 – 0.019)	0.017	-0.052 (-0.174 – 0.070)	0.405
SJC28	-0.002 (-0.047 – 0.043)	0.023	-0.006 (-0.119 – 0.107)	0.917
General health	0.007 (0.002 – 0.011)	0.002	0.179 (0.060 – 0.297)	0.003
Age: 55-65 years	0.073 (-0.137 – 0.283)	0.107	0.041 (-0.077 – 0.159)	0.495
Age: >65 years	0.292 (0.081 – 0.504)	0.107	0.165 (0.046 – 0.284)	0.007
Overweight	-0.257 (-0.494 – -0.020)	0.120	-0.154 (-0.296 - -0.012)	0.033
Normal weight	-0.328 (-0.571 – -0.085)	0.124	-0.192 (-0.335 - -0.050)	0.008

*ESR = erythrocyte sedimentation rate, CRP = C-Reactive Protein, TJC28 = tender joint count in 28-joints, SJC28 = swollen joint count in 28-joints. CI = confidence interval. R^2^ ESR-model: 15.6%, R^2^ CRP model: 6.9%.

^#^After 1 year, sex was no longer significantly associated with CRP levels (p = 0.855). Consequently, this factor is no longer included in the CRP model.

## Discussion

In this study, age and sex were independently associated with the levels of both acute phase reactants in early RA, although the effects appeared to be strongest on the ESR. These results emphasize the need to take these external factors into account when interpreting disease activity in patients with early RA. Because the acute phase reactants tend to increase with age, independent of other core measures of disease activity, the disease activity of older-aged patients might be overestimated. Also, ESR values are more likely to be elevated in women than in men, whereas the opposite appears to be the case for CRP.

Although no significant interactions were found between age and sex, the gradually decreasing differences in ESR and CRP between male and female patients with age are consistent with findings from Radovits et al. [15]. Furthermore, the finding that the ESR tends to be more elevated in women than in men is consistent with the results of previous studies [11–15]. However, inconclusive results have been reported on sex differences in CRP levels. Where Lee et al. [23] found CRP values to be higher in males than in females, other studies have reported the opposite [22, 24]. Ethnic differences across population samples (including genetic variations, diet, and lifestyle) [13, 23] and the use of different measurement methods for determining the CRP levels may possibly explain these diverse results. Since the patient population of this present study is probably more similar to the France and US populations of Piéroni et al. [22] and Lakoski et al. [24], respectively, than the Korean population examined by Lee et al. [23], it was expected to find higher CRP levels in females as well. Nevertheless, the exact underlying mechanisms behind these dependencies remain unknown and all these previous studies were conducted in healthy community populations instead of in samples with active disease. Perhaps the underlying processes of early RA, certain lifestyle differences, the higher prevalence of obesity in the women, hormonal factors, or differences in metabolic risk factor [14, 15, 23] might (partly) explain the higher baseline CRP levels in men in this study or the diminishing association between sex and CRP after 1 year. However, this warrants further research.

Although one recent study in general RA patients concluded that age was unrelated to CRP [15], Ranganath et al. [16] did find significant associations of age with both inflammatory markers in early RA. Likewise, the increasing inflammatory marker levels with age are consistent with a wide variety of other studies [11–14, 16, 17, 21–23]. Like sex differences, the increasing ESR and CRP levels with age might be explained by certain immunological or hematological changes and, for instance, hormonal changes in women who reach the menopause [14, 15, 17]. Furthermore, it has been suggested that RA in older aged people might be more severe than in the young [15]. Supplementary post-hoc analyses did indeed show significantly more swollen joints, higher inflammatory values, and a more active disease according to the DAS28-ESR score in the two highest age groups (55-65 and >65 years old) compared to the youngest group (<55 years old).

In contrast to previous studies, no independent association between BMI and acute phase reactants was found at baseline. Perhaps the proportion of obese patients in this study was too small to detect these effects (15.2% of the men and 21.7% of the women). Likewise, only 8 patients were underweighted with a BMI <18.5 kg/m^2^ and only 4 patients had morbid overweight with a BMI >40 kg/m^2^. However, BMI did become significantly associated with ESR and CRP after 1 year, which might indicate its possible importance at later stages of the disease. It has been suggested that higher BMI levels (i.e. overweight or obese) promote a higher secretion of interleukin-6 (IL-6), a pro-inflammatory cytokine, regulating the secretion of the acute phase reactants [21–23, 26]. A factor which might play a role in this association is the patient’s physical activity [23, 25] given its potential positive effects on the body mass index. Vigorous physical activity might prevent the accumulation of abdominal fat which, consequently, may result in a reduced IL-6 production and lower levels of the acute phase reactants [23, 32]. Taking this one step further, this might also explain why the relation with sex dissipated in the CRP model. If men were more active than women, this might have caused their CRP levels to drop significantly more, reducing any sex effects [32]. However, a thorough understanding of the relationship between physical activity and BMI or the acute phase reactants remains uncertain and warrants further research.

A possible limitation of the current study might be the exclusion of other potential confounding variables, such as certain lifestyle factors (e.g. smoking, physical activity), dietary patterns, or medication use (e.g. estrogen, steroids, or NSAIDs) [21–23, 25, 33]. To study the effects of these variables, further research is needed. Till then, the results of this study should be interpreted with caution. Furthermore, it is a notable finding that the acute phase reactants as well as the number of affected joints were relatively low in the recruited patient sample, even though they were not in remission (DAS28 > 2.6). Yet this is not very surprising given the early stage of their disease. The disease might still be developing when the patient comes to visit the clinic, but enough symptoms may be present for classification and for inclusion in the cohort. Still, this could limit the generalizability of the results towards other populations with more active disease. Finally, even though the proportion of females in this study (61%) might seem relatively low and the average age of the patients (57 years old) might seem relatively high for the early onset of RA, these numbers do correspond to previous results of (Dutch) early RA populations [34–36]. Nevertheless, the results of this study might not be generalizable to populations with a different composition.

## Conclusions

In conclusion, these results suggest that caution should be taken when assessing disease activity in early RA because the ESR and CRP levels are both influenced by non-inflammatory factors like age and sex. Consequently, disease activity might be either overestimated or underestimated. Since the CRP appeared to be less sensitive to external factors this might be designated as the preferred measure, which is consistent with results from Radovits et al. [15] and Crowson et al. [20]. However, one should keep in mind that there might be other confounding factors that were not included in this study (e.g. NSAIDs or corticosteroids), but which may affect the inflammatory markers as well, potentially making the use of the ESR equally acceptable. It could also be argued to develop modified DAS28 scores, including an adaptation for age and sex because these are two risk factors that cannot be modified. Miller et al. [11] already proposed a simple formula for calculating age-adjusted ESR values. However, this was several decades ago, so supplementary research on the possible incorporation of sex and age adjustments in the DAS28 formula is recommended. Another solution might be to specify age and sex specific thresholds of current disease activity scores in order to make them comparable across patient subgroups of different age and sex. Yet to determine the values of such thresholds, further research is required.

## Abbreviations

RA: Rheumatoid arthritis
ESR: Erythrocyte sedimentation rate
CRP: C-reactive protein
DAS28: 28-joint Disease Activity Score
TJC28: 28-tender joint count
SJC28: 28-swollen joint count
GH: General health
BMI: Body mass index
HAQ: Health Assessment Questionnaire
SF-36: 36-item Short Form Health Survey.
